# Performance Assessment of Ultrascaled Vacuum Gate Dielectric MoS_2_ Field-Effect Transistors: Avoiding Oxide Instabilities in Radiation Environments

**DOI:** 10.3390/mi16010033

**Published:** 2024-12-28

**Authors:** Khalil Tamersit, Abdellah Kouzou, José Rodriguez, Mohamed Abdelrahem

**Affiliations:** 1 National School of Nanoscience and Nanotechnology, Abdelhafid Ihaddaden Scientific and Technological Hub, Sidi Abdellah, Algiers 16000, Algeria; 2 Laboratory of Inverse Problems, Modeling, Information and Systems (PIMIS), Université 8 Mai 1945 Guelma, Guelma 24000, Algeria; 3 Applied Automation and Industrial Diagnosis Laboratory (LAADI), Faculty of Science and Technology, Djelfa University, Djelfa 17000, Algeria; kouzouabdellah@ieee.org; 4 High-Power Converter Systems (HLU), Technical University of Munich (TUM), 80333 Munich, Germany; 5 Center for Energy Transition, Universidad San Sebastián, Santiago 8420524, Chile; jose.rodriguezp@uss.cl; 6 Electrical Engineering Department, Faculty of Engineering, Assiut University, Assiut 71516, Egypt

**Keywords:** transition metal dichalcogenide (TMD), vacuum gate dielectric (VGD), field-effect transistors (FETs), non-equilibrium green’s function (NEGF), subthreshold swing (SS), current ratio, switching, oxide instabilities

## Abstract

Gate dielectrics are essential components in nanoscale field-effect transistors (FETs), but they often face significant instabilities when exposed to harsh environments, such as radioactive conditions, leading to unreliable device performance. In this paper, we evaluate the performance of ultrascaled transition metal dichalcogenide (TMD) FETs equipped with vacuum gate dielectric (VGD) as a means to circumvent oxide-related instabilities. The nanodevice is computationally assessed using a quantum simulation approach based on the self-consistent solutions of the Poisson equation and the quantum transport equation under the ballistic transport regime. The performance evaluation includes analysis of the transfer characteristics, subthreshold swing, on-state and off-state currents, current ratio, and scaling limits. Simulation results demonstrate that the investigated VGD TMD FET, featuring a gate-all-around (GAA) configuration, a TMD-based channel, and a thin vacuum gate dielectric, collectively compensates for the low dielectric constant of the VGD, enabling exceptional electrostatic control. This combination ensures superior switching performance in the ultrascaled regime, achieving a high current ratio and steep subthreshold characteristics. These findings position the GAA-VGD TMD FET as a promising candidate for advanced radiation-hardened nanoelectronics.

## 1. Introduction

The advancement of technology has enabled remarkable progress in designing devices that operate reliably in harsh environments, such as radioactive, high-temperature, and corrosive conditions [1,2,3]. These advancements are crucial for applications in space exploration, nuclear energy, and medical technologies, where environmental stressors can severely impact device functionality [1,2,3,4,5]. Among the key challenges in such environments is the development of robust electronic and nanoelectronic systems capable of maintaining reliable performance under extreme conditions [4,5,6].

Radiation-immune nanoelectronics, in particular, have garnered significant attention due to their ability to withstand the detrimental effects of ionizing radiation [6,7]. Such devices are indispensable in applications requiring high levels of reliability, including spacecraft, nuclear reactors, and military systems [1,2,3,4,5,6,7]. However, achieving radiation immunity at the nanoscale remains a formidable challenge, primarily because conventional oxide-based gate dielectrics in field-effect transistors (FETs) are highly susceptible to radiation-induced instabilities [8,9,10]. These instabilities include charge trapping, interface state generation, and oxide breakdown, all of which degrade the device’s electrical performance and reliability [5,8].

Vacuum technology has emerged as a promising alternative to conventional dielectrics, offering solutions to many of these issues [11,12,13,14,15,16,17,18,19,20]. In particular, the use of vacuum gate dielectric (VGD) and vacuum channels in FETs eliminates the susceptibility to radiation-induced charge trapping and interface states, enabling greater stability and durability in extreme conditions [7,12,20]. However, the low dielectric constant of a vacuum presents a significant challenge to electrostatic control, especially in nanoscale devices.

To address this issue, advanced techniques such as the gate-all-around (GAA) configuration [21], the incorporation of two-dimensional (2D) materials [22], and the engineering of coupling vacuum capacitance [12,20] can be proposed. These approaches can synergistically enhance the electrostatic gating, ensuring efficient carrier transport and reducing short-channel effects in FETs equipped with vacuum gate dielectric. In this work, we computationally evaluate the performance of ultrascaled transition metal dichalcogenide (TMD) FETs featuring a vacuum gate dielectric, a gate-all-around configuration, and a 2D monolayer TMD channel. Using a quantum simulation approach [23], we assess the impact of these design features on the device’s switching performance, scaling limits, and suitability for radiation-hardened applications.

The structure of this paper is as follows: Section II introduces the device structure, detailing the design considerations and material choices. Section III describes the quantum simulation approach, including the self-consistent solution of the Poisson and quantum transport equations. Section IV presents the results and discusses the performance metrics and their implications for nanoelectronic design. Finally, Section V concludes this paper.

## 2. Nanodevice Structure and Radiation-Induced Oxide Instabilities

**Figure 1:**
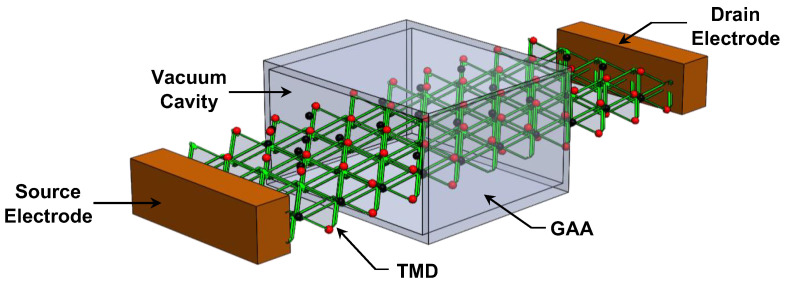
Three-dimensional (3D) structure of ultrascaled gate-all-around vacuum gate dielectric transition metal dichalcogenide field-effect transistor (GAA-VGD TMDFET).

Figure 1 illustrates a perspective view of the gate-all-around vacuum gate dielectric transition metal dichalcogenide field-effect transistor under investigation. The device features a monolayer TMD-based channel with an ultrascaled width and an n-i-n doping profile, comprising an intrinsic channel beneath the gate of length, L_G_, and n-type doped source and drain regions, each of length L_S_ and L_D_, respectively, connected to the source and drain electrodes. A gate-all-around configuration has been adopted to ensure excellent electrostatic control [24,25] while compensating for the low dielectric constant of the vacuum. The three-dimensional structure also highlights the vacuum gate dielectric paradigm, consisting of a vacuum cavity surrounding the intrinsic channel. In the case of considering oxide gate dielectric [26], such as SiO_2_, in radioactive environments, high-energy radiation generates electron–hole pairs within oxide layers; some recombine, while others separate depending on the biasing conditions. This process leads to a buildup of radiation-induced charges, causing device degradation [5,8,26,27,28]. By replacing the oxide layer with a vacuum dielectric, the gate leakage current and the radiation-induced issues are effectively eliminated. Furthermore, the TMD channel material is assumed to have negligible susceptibility to radiation damage due to its two-dimensional nature, which minimizes bulk defects commonly found in three-dimensional semiconductors [29,30]. However, the low dielectric constant of vacuum (ε_VAC_ = 1) reduces the gate’s electrostatic control over the channel carriers [12,20]. This is exactly the motivation of the present investigation, which focuses on the performance analysis of VGD-based TMD transistors to overcome these limitations and achieve robust performance in harsh environments.

## 3. Quantum Simulation Approach

The analysis of the gate-all-around vacuum gate dielectric (GAA-VGD) TMD field-effect transistor leverages an advanced and powerful quantum simulation methodology. This approach integrates the Poisson equation with the Schrödinger equations within the framework of the non-equilibrium Green’s function (NEGF) formalism [31,32,33,34,35]. The sub-10 nm channel lengths allow the assumption of ballistic transport, omitting scattering effects [23,36,37,38]. The computational representation of TMD monolayers relies on an effective mass Hamiltonian, which is a part of the retarded Green’s function, as [31]

(1) $$G(E)={\left[(E+i0^{+})I-H-\sum_{S}-\sum_{D}\right]}^{-1}$$

where *H* is the Hamiltonian matrix, *E* represents the energy, *I* is the identity matrix, 0^+^ indicates a small positive value approaching zero, and *Σ_S_*_(*D*)_ refers to the self-energy of the source (drain) contact [31]. The local density of states *D_S_*_(*D*)_ is subsequently determined by the following expression [31]:

(2) $$D_{S(D)}=G\Gamma_{S(D)}G^{\text{†}}$$

where $\Gamma_{S(D)}=i(\Sigma_{S(D)}-\Sigma_{S(D)}^{\text{†}})$ is the broadening function pertaining to the source (drain) contact. From an electrostatic perspective, the Poisson equation was solved, as given by the following equation [24]:

∇ · (ε_r_∇ V) = ρ/ε_0_ (3)

where *ε*_r_ denotes the relative permittivity of the materials, *V* is the electrostatic potential, ρ stands for the charge density, and ε_0_ represents the permittivity of free space. Within the framework of the vacuum gate dielectric (VGD) paradigm, the dielectric constant in the vacuum region is designated as *ε_VAC_* = 1. Neumann boundary conditions are applied to all external surfaces except at the GAA nodes, where Dirichlet boundary conditions are implemented. After the self-consistent NEGF-Poisson system converges, the drain current can be computed as outlined in [36,37,38]. Further details on the NEGF simulation methodology can be found in previous computational studies [39,40,41,42].

## 4. Results and Discussions

**Figure 2:**
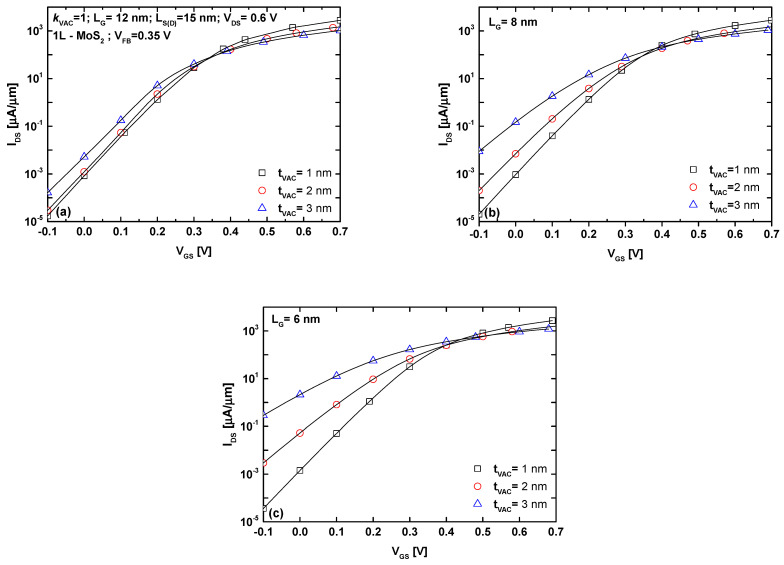
I_DS_-V_GS_ transfer characteristics of the sub-12 nm GAA-VGD MoS_2_ FET. (**a**) L_G_ = 12 nm; (**b**) L_G_ = 8 nm; (**c**) L_G_ = 6 nm.

Figure 2 illustrates the behavior of the GAA-VGD MoS_2_ FET in terms of transfer characteristics, considering gate downscaling. A gate length of 12 nm is analyzed in Figure 2a to evaluate the device under the 2037 IRDS requirements [42]. The physical and geometrical parameters are shown in the same figure. It is evident that the device exhibits acceptable switching behavior while considering VGD, with a subthreshold swing close to the thermal limit of 60 mV/dec, which is below the IRDS requirement [43]. This behavior is mainly attributed to the excellent GAA electrostatics on the TMD 2D materials [23]. Additionally, a slight decrease in the vacuum gate dielectric (VGD) thickness marginally increases the on-state current while reducing the off-state current. For a gate length of 8 nm, as shown in Figure 2b, the impact of VGD thickness becomes more significant on the off-state current and subthreshold swing. Specifically, a VGD thickness of 1 nm leads to substantial improvements in I_OFF_ and SS compared to thicker VGD layers. By further scaling the gate length to 6 nm, as shown in Figure 2c, the improvements in off-current and subthreshold swing become more pronounced. Notably, when the VGD thickness is reduced from 3 nm to 1 nm, I_OFF_ decreases by approximately four orders of magnitude, leading to steeper SS. Inspection of the same figure reveals that reducing the VGD thickness has negligible impact on the on-current for all ranges of gate length. It is worth noting that Figure 2 provides implicit information about the device’s behavior when using a low-k dielectric (e.g., SiO_2_), which improves the coupling capacitance in comparison to a VGD device.

**Figure 3:**
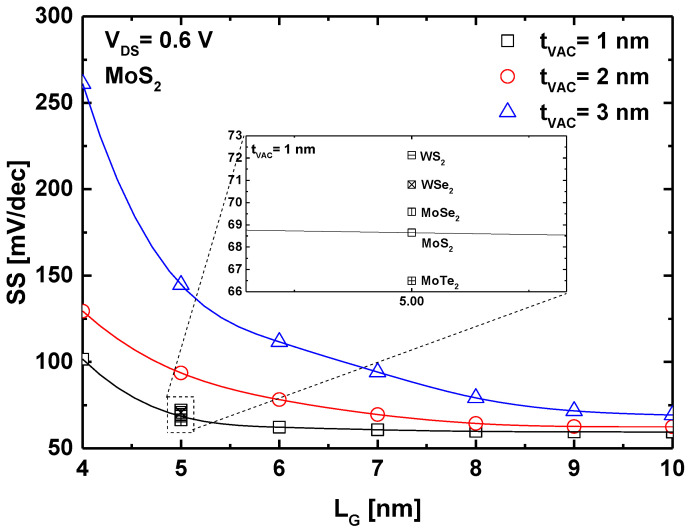
Subthreshold swing versus gate length of sub-10 nm GAA-VGD TMD FET for different VGD thickness.

Figure 3 illustrates the behavior of the subthreshold swing with sub-10 nm gate downscaling for the GAA-VGD TMD FET under investigation. The figure clearly shows an increase in the subthreshold swing as the gate length decreases for three different values of vacuum gate dielectric (VGD) thickness. Notably, reducing the VGD thickness significantly improves the subthreshold swing, with a remarkable improvement observed for sub-5 nm gate lengths, where the SS factor decreases from 260 mV/dec to 100 mV/dec for a 4 nm gate length. This result indicates that for ultrascaled GAA-VGD TMD FETs, employing a thinner vacuum space as the gate dielectric is effective in mitigating SCEs while improving the device immunity. It also highlights the potential of such nanodevices based on the VGD paradigm for operation in harsh environments, where oxide-induced fluctuations can be significantly detrimental. The inset in Figure 3 further reveals that vacuum FETs based on other TMD materials exhibit close SS values, with MoTe_2_-based VGD FETs demonstrating the lowest swing factor. Additionally, MoX_2_-based VGD FETs show better SS performance compared to WX_2_-based VGD FETs. This can be logically attributed to the higher effective carrier mass in MoX_2_, which suppresses direct source-to-drain tunneling in the aggressively scaled domain. It is worth noting that this result aligns with previously reported findings on TMD FETs with solid gate dielectrics [23].

**Figure 4:**
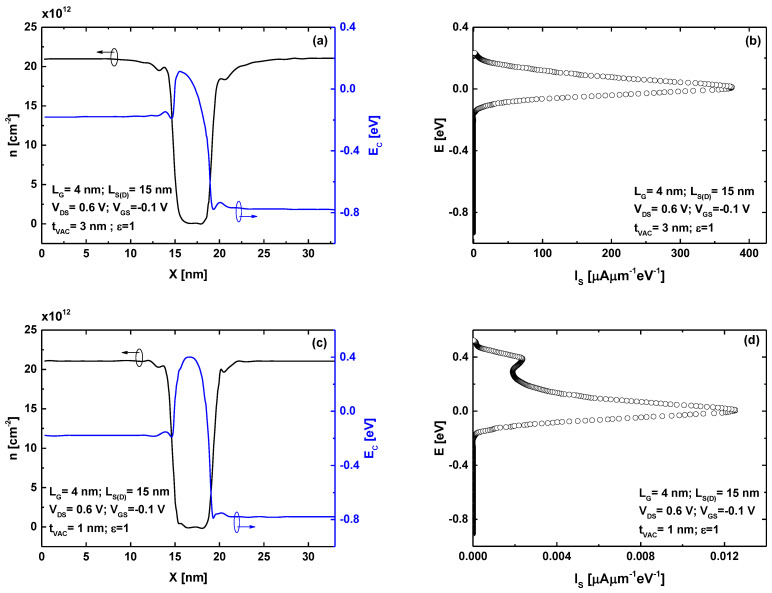
Band profile and charge density at the same off-state bias for (**a**) t_VAC_ = 3 nm and (**c**) t_VAC_ = 1 nm. The corresponding current spectrum for (**b**) t_VAC_ = 3 nm and (**d**) t_VAC_ = 1 nm.

To understand the significant improvement in the off-state current and subthreshold swing observed at the ultrascaled regime using a thin vacuum gate dielectric, in Figure 4, we present the potential profile, charge density, and corresponding current spectrum for a 4 nm gate length, considering two different vacuum gate dielectric thicknesses, namely 1 nm and 3 nm. Figure 4a shows the band profile and charge density of the GAA VGD-TMD FET in the off-state for a VGD thickness of 3 nm. Figure 4b displays the corresponding current spectrum for the same VGD thickness. It is evident that the device exhibits thermionic emission and significant direct source-to-drain tunneling (DSDT) at the top of the barrier. This behavior arises from the narrow potential barrier, which is attributed to the vacuum coupling capacitance, leading to poor electrostatic control. Figure 4c demonstrates that reducing the VGD thickness from 3 nm to 1 nm enhances electrostatic control, resulting in an increased potential barrier. This reduction also influences the charge density distribution, which shows an expansion beneath the gate and, thus, a reduction in channel charge density. Figure 4d highlights the corresponding decrease in the current spectrum in the off-state, attributed to the improved electrostatic control achieved by reducing the VGD thickness, as evidenced in the comparison between Figure 4a,c. Additionally, the current spectrum reveals two distinct components: a thermionic peak tangent to the top of the potential barrier and a DSDT peak through the potential barrier.

**Figure 5:**
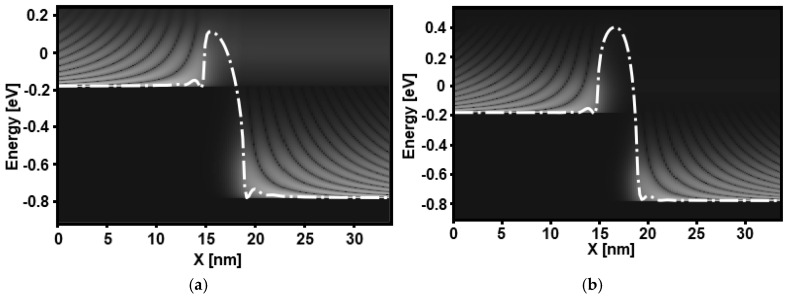
Local density of states at V_GS_ = −0.1 V and V_DS_ = 0.6 V considering 4 nm gate length. (**a**) t_VAC_ = 3 nm and (**b**) t_VAC_ = 1 nm.

Figure 5 illustrates the local density of states extracted in the subthreshold regime for an ultrascaled VGD-TMD device, considering two different VGD thicknesses. In both cases, oscillation patterns due to quantum reflections are clearly observed. In Figure 5a, the poor electrostatic gating, attributed to the small VGD coupling capacitance, permits significant DSDT components through the potential barrier. However, as shown in Figure 5b, reducing the VGD thickness enhances electrostatic gating, resulting in a longer and wider potential barrier, which significantly reduces the DSDT components.

**Figure 6:**
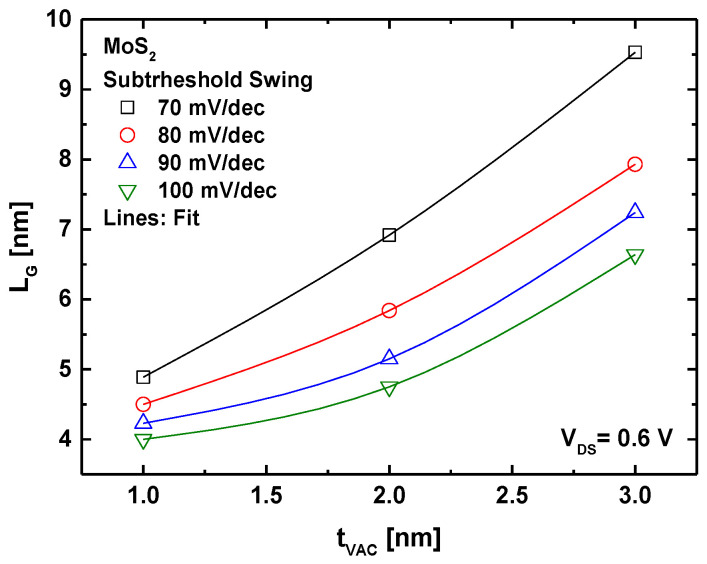
Graphical abacus analyzing the subthreshold swing and downscaling of GAA-VGD MoS_2_ FET.

Figure 6 presents a graphical analysis of the downscaling behavior of the proposed VGD TMD transistor, targeting various sub-100 mV/dec subthreshold swing values. It is observed that a thinner VGD thickness (i.e., 1 nm) enables SS values between 70 and 100 mV/dec with sub-5 nm gate lengths, where achieving a lower SS requires an increase in gate length in the sub-5 nm range. This highlights the effective control of carrier transport in MoS_2_ FETs when utilizing a thin VGD (compensating for the low vacuum dielectric constant) in combination with the gate-all-around configuration that provides excellent electrostatic gating. The figure further shows that a thicker vacuum gate dielectric necessitates longer gate lengths to achieve the desired SS values. However, for all targeted sub-100 mV/dec SS values, gate lengths remain below 10 nm, demonstrating that the GAA VGD-TMD FET is a promising candidate for advanced radioactive environments such as medical, nuclear, and space applications, where the use of oxide gate dielectrics is not recommended. It is worth noting that VGD FETs based on other TMD materials, including MoSe_2_, MoTe_2_, WS_2_, and WSe_2_, have also been evaluated, showing similar downscaling behavior. Among these, MoX_2_-based devices exhibit improved subthreshold performance due to the higher effective mass of the carrier, which reduces direct source-to-drain tunneling.

**Figure 7:**
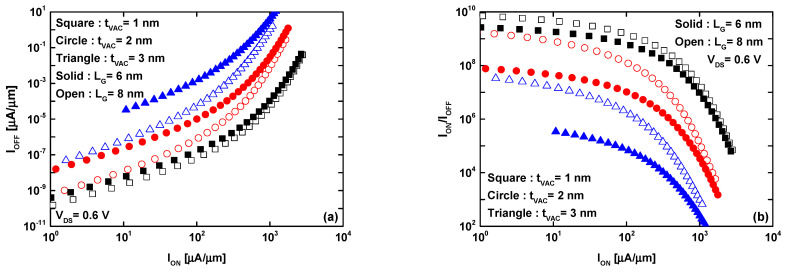
(**a**) I_OFF_ versus I_ON_ and (**b**) I_ON_/I_OFF_ current ratio versus I_ON_ for L_G_ = 6 and 8 nm and t_VAC_ = 1, 2, and 3 nm.

Figure 7 is drawn from the transfer characteristics of Figure 2 by considering a window with a right (left) extremity equal to V_GS-ON_ (V_GS-OFF_ = V_GS-ON_ − V_DD_). Every time we shift the V_DD_ (here, equal to V_DS_ = 0.6 V) window by a given step (e.g., 0.05 V), we extract I_DS-OFF_ and I_DS-ON_, which correspond to V_GS-OFF_ and V_GS-ON_, respectively, until sweeping the entire V_GS_ range. Note that this plot allows us to highlight the possible current ratios versus off- or on-current, as well as to estimate the maximum reachable current ratio.

Figure 7a illustrates the off-state current as a function of the on-state current, considering different sub-3 nm VGD thicknesses and sub-10 nm gate lengths. By fixing a shared on-state current as the desired value, it is evident that using a thinner VGD thickness is advantageous for achieving a low off-state current by improving electrostatic control. Notably, there is no substantial difference in the recorded off-state currents for VGD-TMD FETs with L_G_ = 6 nm and L_G_ = 8 nm when employing a 1 nm VGD thickness. However, the difference in off-state current becomes significant with a thicker vacuum gate dielectric. In such cases, longer gate lengths are highly beneficial in reducing off-state current and mitigating short-channel effects, as shown in the same figure for FETs with a 3 nm VGD thickness. A similar trend is observed when plotting the I_ON_/I_OFF_ current ratio as a function of on-state current, as shown in Figure 7b. Thinner VGD thicknesses are suitable for achieving a high current ratio, while the difference in I_ON_/I_OFF_ current ratio across the two different sub-10 nm gate lengths becomes more pronounced as the coupling vacuum capacitance decreases with increasing VGD thickness. These results highlight that the TMD-based channel, in combination with the vacuum gate dielectric paradigm and gate-all-around configuration, makes sub-10 nm GAA VGD-TMD FETs promising candidates for switching applications in harsh environments that include advanced medical, space, nuclear, and military applications, where radiation-immune nanoelectronics are essential.

For advanced investigations, the studied vacuum gate dielectric transition metal dichalcogenide transistors can be utilized to evaluate fundamental electronic circuits, such as SRAM and logic gates, under radioactive conditions, with particular attention to the behavior of interconnects [44,45,46,47,48]. Furthermore, the combination of electrostatic doping with the vacuum gate dielectric paradigm presents a pathway to realizing ultrascaled gate-all-around (GAA) vacuum gate dielectric dopingless transition metal dichalcogenide (tunnel) field-effect transistors [49,50,51,52]. In addition, these devices could also serve as building blocks for more complex systems in harsh environments, such as radiation-tolerant neuromorphic computing platforms or high-performance processors in space and/or medical applications. Additionally, exploring alternative transition metal dichalcogenide materials with higher radiation resilience or integrating hybrid 2D/3D architectures could further enhance their applicability. Future studies could investigate the scalability limits of these transistors and their compatibility with advanced fabrication techniques, paving the way for innovative nanoscale electronic solutions.

## 5. Conclusions

This study highlights the potential of ultrascaled transition metal dichalcogenide (TMD) field-effect transistors (FETs) equipped with vacuum gate dielectric (VGD) for addressing the challenges of oxide instabilities in harsh environments, such as radioactive conditions. Through quantum simulations, the proposed GAA-VGD TMD FET demonstrated superior electrostatic control and high switching performance, characterized by a steep subthreshold swing and a good current ratio, even in the ultrascaled regime. These findings confirm the effectiveness of combining a gate-all-around (GAA) configuration, a TMD-based channel, and a thin vacuum gate dielectric in achieving reliable and high-performance nanoelectronics for extreme applications.

## Data Availability

The data that support the findings of this study are available from the first corresponding author (K.T.) upon reasonable request.
